# Preparation of ZnS@In_2_S_3_ Core@shell Composite for Enhanced Photocatalytic Degradation of Gaseous *o*-Dichlorobenzene under Visible Light

**DOI:** 10.1038/s41598-017-16732-4

**Published:** 2017-11-27

**Authors:** Baojun Liu, Xia Hu, Xinyong Li, Ying Li, Chang Chen, Kwok-ho Lam

**Affiliations:** 1 College of Resource and Environmental Engineering, Guizhou University, Guiyang, 550025 China; 2 Department of Electrical Engineering, The Hong Kong Polytechnic University, Hung Hom, Kowloon, Hong Kong, China; 3 State Key Laboratory of Fine Chemicals, Key Laboratory of Industrial Ecology and Environmental Engineering (MOE), School of Environmental Science and Technology, Dalian University of Technology, Dalian, 116024 China

## Abstract

In this study, novel ZnS@In_2_S_3_ core@shell hollow nanospheres were fabricated by a facile refluxing method for the first time, and the formation mechanism of hollow structure with interior architecture was discussed based on ion-exchange Ostwald ripening. As the photocatalytic material for degradation of gaseous *o*-Dichlorobenzene (*o*-DCB), the as-synthesized core@shell hollow nanospheres were found to show significantly enhanced catalytic performance for effective separation of photo-generated charges. Moreover, the mechanisms of enhanced activity were elucidated by band alignment and unique configuration. Such photocatalyst would meet the demands for the control of persistent organic pollutant (POPs) in the atmospheric environment.

## Introduction

Zinc sulphide (ZnS) semiconductor, as an important II-VI binary chalcogenide, has attracted much attention due to its wide potential applications in many fields^1–3^. For example, ZnS materials possess photocatalytic properties for pollutant control and photosynthesis process^4–6^. However, its wide band gap and high recombination rate of photo-generated charges often lead to low quantum yield in the photocatalytic reactions. To address the key issue of low quantum yield, many methods, such as noble metal deposition^7,8^, element doping^9^ and semiconductor coupling^10,11^, have been extensively studied and applied in recent years^12–15^. Among these approaches, coupling with other semiconductors for forming the composites is one of the most effective ways to improve the separation efficiency of photo-generated charges, which could also take full advantage of solar spectrum, compared with the individual material^16,17^.

At present, indium sulphide (In_2_S_3_) material, a III-VI group sulphide, is a semiconductor with a bandgap of 2.0–2.3 eV and would show excellent optical property, especially, a wide response to the solar spectrum^18,19^. More importantly, the conduction band (CB) of In_2_S_3_ is composed of d, s and p orbitals, while the valence band (VB) consists of S 3p orbitals, which are much more negative than O 2p orbitals^3^. The characteristics of band edges could lead to the reduction reactions more available. In spite of these advantages of In_2_S_3_, it is obvious that the strong light-etching phenomenon and low charge-migration efficiency would prevent it from practical applications as an individual component^20,21^. Thus, the coupling of In_2_S_3_ with ZnS material for forming heterogeneous structures should be beneficial for effective separation and transfer of photo-induced carriers. In addition, since the hollow structure can modulate the index of refraction, enhance intensity of light scattering, and provide more active sites for substances adsorption and facilitate diffusion between substances and materials^22–24^, the hollow-structured catalysts could improve the photocatalytic efficiency significantly. Generally, the shells of hollow structure are accumulated by a large quantity of nanoparticles, which could leave a large number of porous channels in the framework, so oxygen and reactants could permeate through the shells for much efficient reactions^23,25,26^.

Based on the above analysis, ZnS@In_2_S_3_ core@shell hollow nanospheres have been proposed in this work as high-efficient hollow composite nanomaterials for photocatalytic applications. Herein, the ZnS@In_2_S_3_ core@shell hollow nanospheres have been prepared by an anion-exchange reaction for the first time and then applied to photocatalytic degradation of gaseous *o*-Dichlorobenzene (*o*-DCB) under visible-light irradiation. *o*-DCB, as one of the typical persistent organic pollutants (POPs), has raised wide attention for their carcinogenicity, high toxicity and bioaccumulation in the environment^27,28^. It is obvious that the coupling of ZnS with In_2_S_3_ is a promising strategy to enhance the photocatalytic activity for pollutant elimination. Furthermore, the enhancement mechanism of photocatalytic performance for the ZnS@In_2_S_3_ core@shell hollow spheres has been discussed in detail.

## Results and Discussion

### Morphology and Formation Mechanism Analysis

**Figure 1:**
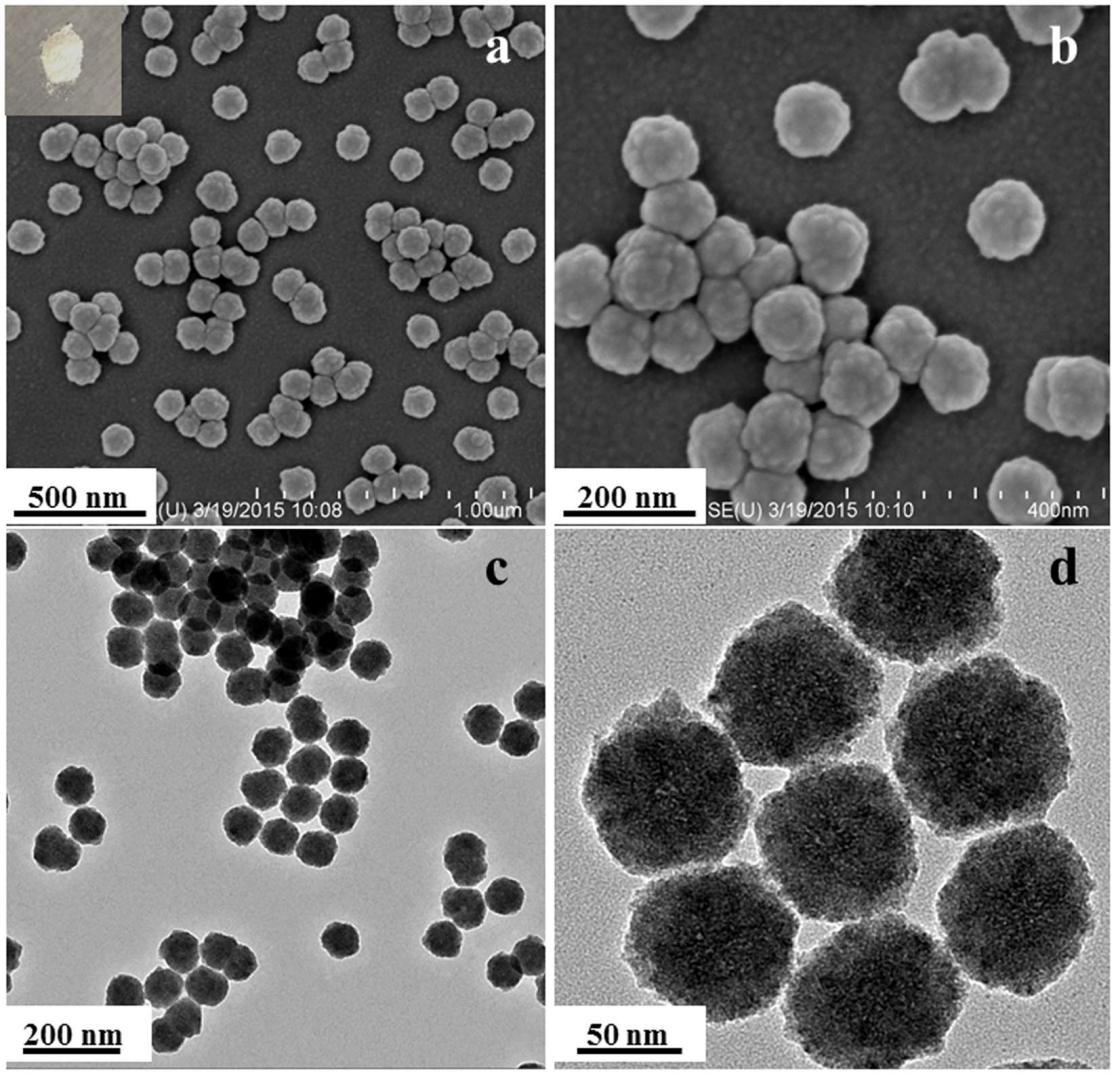
(**a,b**) SEM, (inset of (**a**)) photograph, and (**c,d**) TEM images of pure ZnS spheres.

The morphology and internal structure of the ZnS sample were investigated by field-emission scanning electron microscopy (FESEM) and transmission electron microscopy (TEM). As depicted in Fig. 1a, the ZnS spheres show no evident aggregation with the particle size of about 100 nm, and possess the coarse surface. In Fig. 1b, it is obvious that the surface of ZnS spheres is piled with small nanoparticles. Figure 1c demonstrates that the interior framework exhibits the solid structure, indicating that the morphology has been well obtained by the refluxing method. Moreover, a closer examination shows the coarse edge of the ZnS sample (Fig. 1d), which is consistent with the above SEM images.

**Figure 2:**
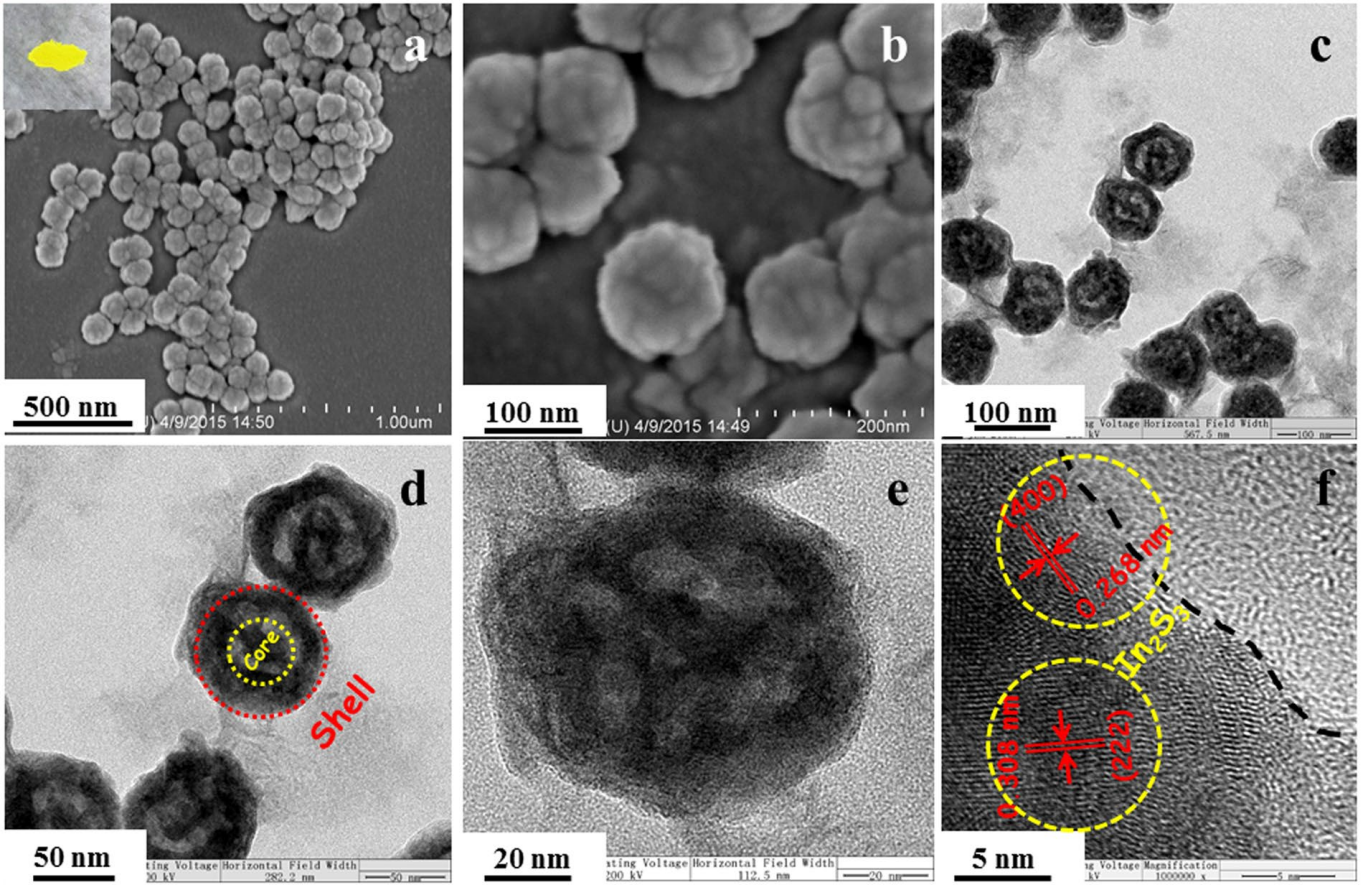
(**a,b**) SEM, (inset of (**a**)) photograph, (**c–e**) TEM and (**f**) HRTEM images of ZnS@In_2_S_3_ core@shell hollow spheres.

After coupled with In_2_S_3_ nanoparticles, the white powder (Fig. 1a) was turned into faint yellow (Fig. 2a). The SEM images of ZnS@In_2_S_3_ composite materials are depicted in Fig. 2a and b. The particle size and morphology of ZnS@In_2_S_3_ are almost similar to those of ZnS, indicating that the uniform spheres have been fabricated and distributed on a large scale. Further investigations of interior structure have been performed through TEM and HRTEM. As shown in Fig. 2c and d, the fabricated ZnS@In_2_S_3_ nanospheres possess the core-shell hollow structure. The specific details are shown in Fig. 2e, and the diameter of the outer shell is about 20 nm. In addition, a typical high-resolution TEM (HRTEM) image from the outer edge displays distinct lattice fringes with the spacing of 0.268 and 0.308 nm corresponding with the (400) and (222) planes of In_2_S_3_, respectively, as shown in Fig. 2f.

**Figure 3:**
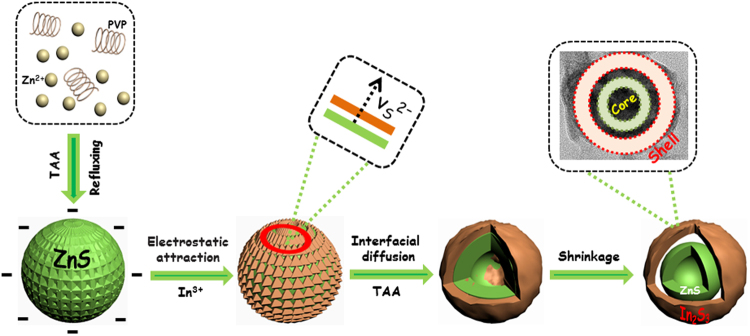
Schematic growth mechanism of the ZnS@In_2_S_3_ core@shell hollow spheres.

Based on the details of SEM and TEM images, one of most possible formation mechanisms of ZnS@In_2_S_3_ core@shell hollow nanospheres is illustrated in Fig. 3. It is known that the pyrrolidon ring of the capping agent PVP possesses the strong interaction with Zn^2+^, which could prevent the agglomeration of ZnS nanoparticles through the repulsive forces among the polyvinyl groups^10^. After added TAA, the S^2−^ ions are released from TAA in the aqueous solution when heated to 100 °C, and then react with Zn^2+^ to form ZnS tiny nanocrystals. To minimize the surface energy, these nanocrystals tend to form into solid spheres by the self-assembled method^29^. As a result, the ZnS solid spheres composed of small grains exhibited greater coarseness. As the surface charge of ZnS materials was negative in aqueous solutions, the In^3+^ ions would attach on the surface of ZnS spheres through the electrostatic attraction. Subsequently, the In_2_S_3_ nanoparticles will be formed on the surface of ZnS crystals via interfacial diffusion^10^, which would form the core@shell ZnS@In_2_S_3_ structure. Obviously, it is believed that the difference of solubility products (*K*_*sp*_) of ZnS (1.6 × 10^−24^) and In_2_S_3_ (6.3 × 10^−36^) is the main driving force for the formation of ZnS@In_2_S_3_ composite^29,30^. At last, after adding TAA again, the S^2−^ concentration in the solution environment is much higher than that in the composites such that the S^2−^ ions of ZnS are hardly to diffuse outside, and so the yolk@shell structure is formed. In the post-heating process, the yolk@shell structure would contract to form the core@shell hollow structure due to the instability.

### Physicochemical property characterization

**Figure 4:**
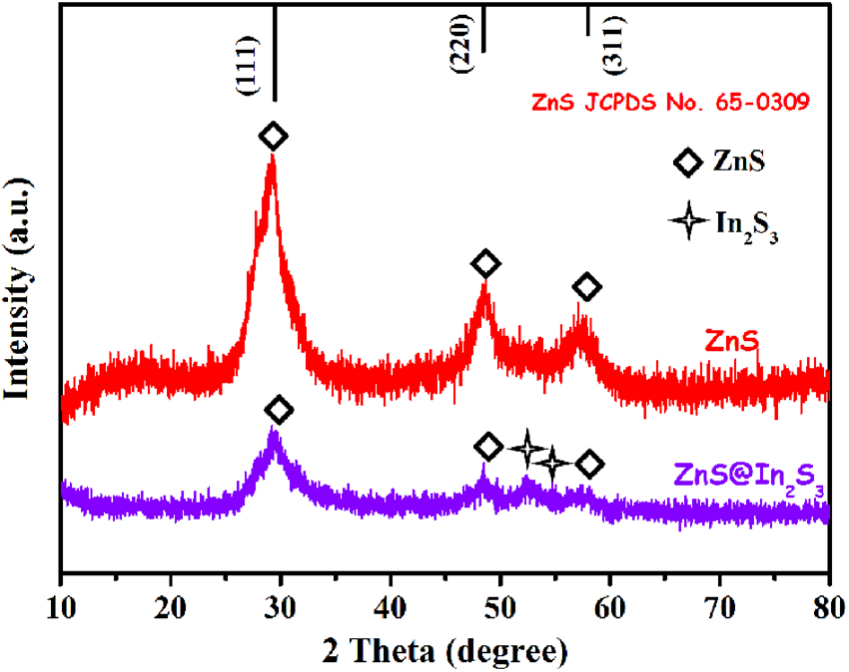
XRD patterns of pure ZnS and ZnS@In_2_S_3_ core@shell hollow spheres.

**Figure 5:**
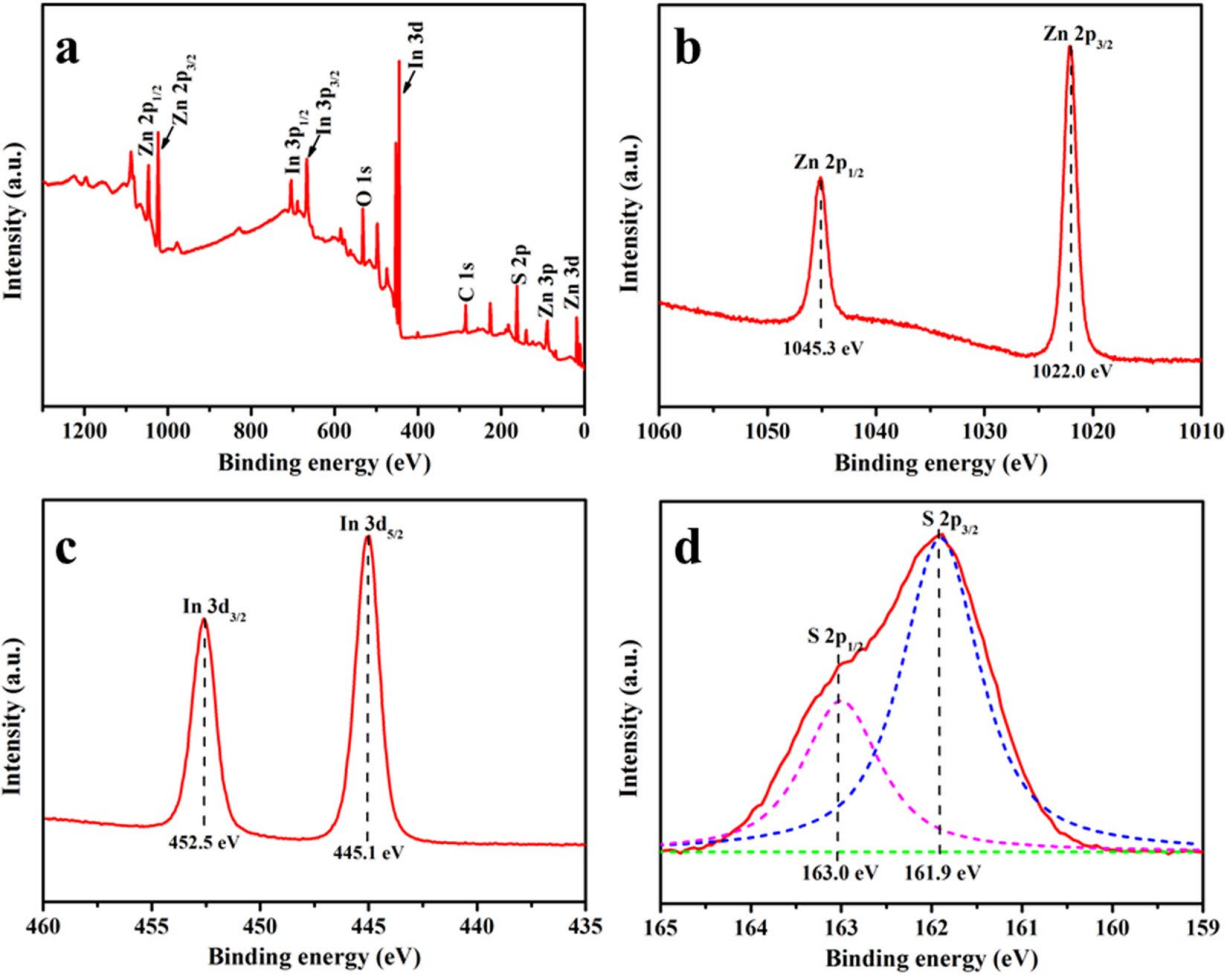
XPS spectra of ZnS@In_2_S_3_ core@shell hollow spheres: (**a**) survey of the sample, (**b**) Zn 2p spectrum, (**c**) In 3d spectrum, (**d**) S 2p spectrum.

The XRD pattern of the ZnS@In_2_S_3_ core@shell spheres is shown in Fig. 4. For the ZnS sample, the characteristic peaks, appearing at 2*θ*: 28.6°, 47.6° and 56.5° would be assigned to the diffraction patterns of (111), (220) and (311) of ZnS cube phase given in JCPDS (65–0309) (space group: F-43m (216)), respectively, and no other peaks of impurities were detected. After coupled with In_2_S_3_ to form the ZnS@In_2_S_3_ structure, the composite exhibits extra diffraction peaks at 2*θ*: 51.6° and 56.0°, which correspond to the (112) and (201) plane reflections of the hexagonal phase In_2_S_3_ given in JCPDS (33-0623) (space group: P-3m1(164)), respectively. This indicates that the core@shell composite structure was fabricated by coupling ZnS with In_2_S_3_. Moreover, in order to further confirm the surface components and chemical states of ZnS@In_2_S_3_ structures, the composite was evaluated by XPS and the results are shown in Fig. 5. From the survey spectrum, it could be seen that the composite material contains Zn, In, S, O, and C elements, and the O element is from the adsorption of H_2_O on the surface of catalyst or residual substances after calcination^31^. In addition, the C element at the binding energy of 284.6 eV is from allogenic substances or referencing spectra^32^. In Fig. 5b, there are two characteristic peaks at 1045.3 eV and 1022.0 eV for Zn 2p_1/2_ and Zn2p_3/2_, respectively, which are assigned to Zn^2+^ ^33^. Meanwhile, the bands at 425.5 eV and 445.1 eV are ascribed to the spectrum of In^3+^ ^34^. Due to its asymmetry of XPS spectrum of S 2p, two evident peaks located at 163.0 eV and 161.9 eV are deconvoluted in Fig. 5d, which are attributed to S^2−^ ^17,34^.

**Figure 6:**
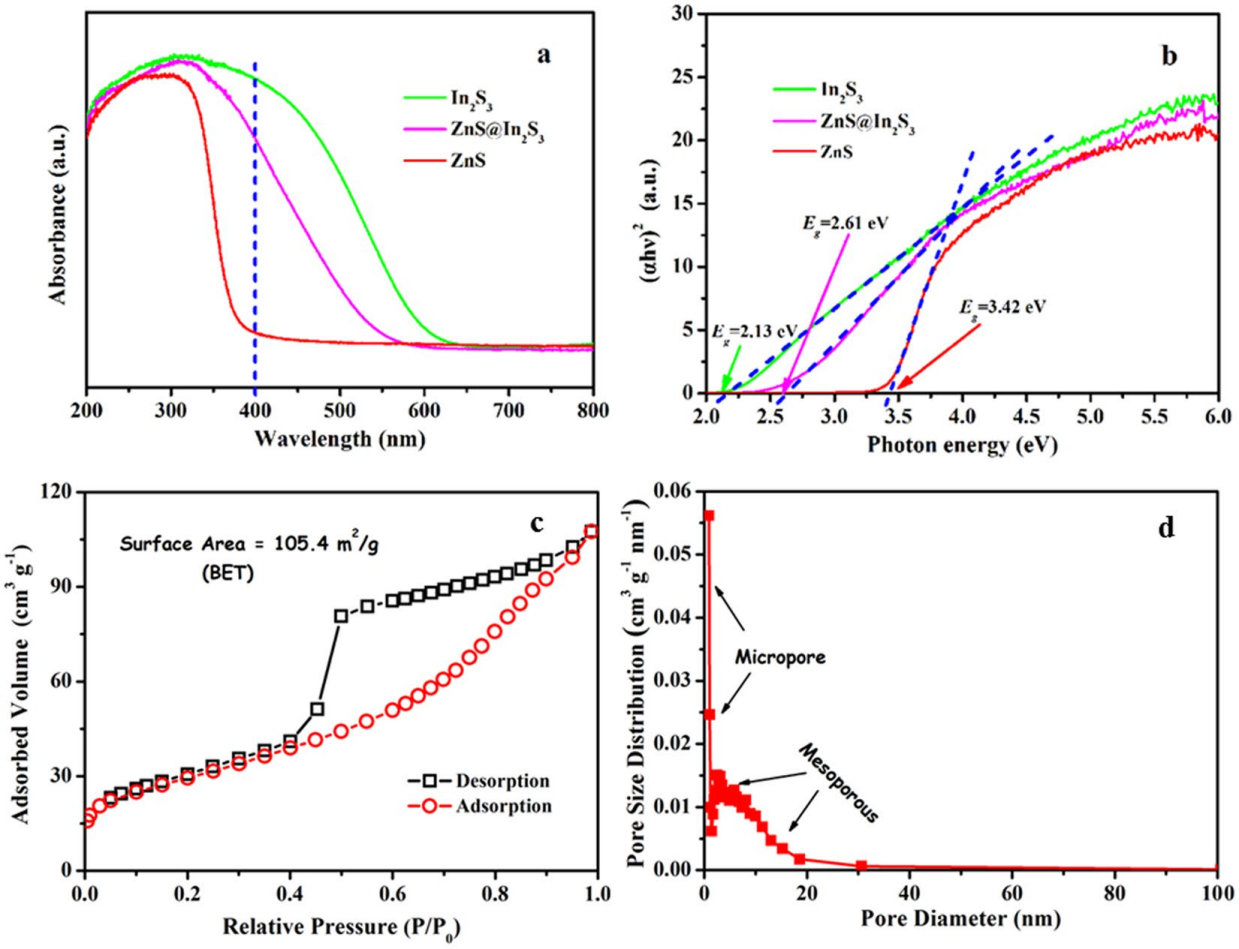
(**a**) UV-vis DRS and (**b**) Tauc’s plot of the obtained samples, respectively; (**c**) N_2_ adsorption/desorption isotherms and (**d**) pore size distribution of the as-prepared ZnS@In_2_S_3_ core@shell hollow spheres.

The optical response properties of the catalytic materials obtained were investigated by UV-vis diffuse reflection spectra (DRS). From Fig. 6a, it is obvious that the pure ZnS shows no evident absorption peak in the region of visible light (400–550 nm), whereas the adsorption edge of the ZnS@In_2_S_3_ composite significantly extend to the visible light region, suggesting that the composite exhibits the enhanced absorption ability under the visible-light irradiation. Furthermore, the band gap *E*_*g*_ values are calculated by estimating the intercept of the tangent at the Tauc’s plots^35^. As depicted in Fig. 6b, the band gap of the ZnS@In_2_S_3_ material was calculated to be 2.61 eV, showing that the electrons could transfer under visible-light illumination. Regarding of the lower recombination rate of photo-generated charges with the addition of In_2_S_3_, the photocurrent and electrochemical impedance spectroscopy (EIS) of ZnS and ZnS@In_2_S_3_ composite were tested under visible light in the electrochemical workstation. As shown in Figure S1a (see Supporting Information), the ZnS@In_2_S_3_ has much higher transient photocurrent than the ZnS that did not respond to visible light, indicating that the ZnS@In_2_S_3_ has higher efficiency of photo-induced charge separation. In addition, Figure S1b exhibits the EIS Nyquist plots of the ZnS and ZnS@In_2_S_3_ electrodes under visible-light illumination in the Na_2_SO_4_ electrolyte. Usually, a smaller radius of the arc on the EIS Nyquist plot represents the faster charge-transfer speed^36,37^. After coupling with In_2_S_3_, the impedance radius of the ZnS electrode reduced significantly, which implies that the ZnS@In_2_S_3_ structure has good response to visible light, so as to effectively improve the separation of photon-generated charges and accelerate the photo-induced charge transfer.

As the pore size and the corresponding specific surface area of ZnS@In_2_S_3_ core@shell spheres play important roles in enhancing the photocatalytic performance, the N_2_ adsorption/desorption isotherms were investigated. As shown in Fig. 6c, the typical IV isotherms with a H1 hysteresis loop were obtained. Besides, the pore size distribution was in the regions of mesoporous and micropore structures (shown in Fig. 6d). Moreover, the specific surface area of the ZnS@In_2_S_3_ core@shell structure was about 105.4 m^2^ g^−1^. Compared to the mixed sulfides (66.2 m^2^ g^−1^), the core-shell structure showed much larger surface, to some extent, which could promote the degradation efficiency.

### The performance and mechanism of degradation gaseous *o*-DCB

**Figure 7:**
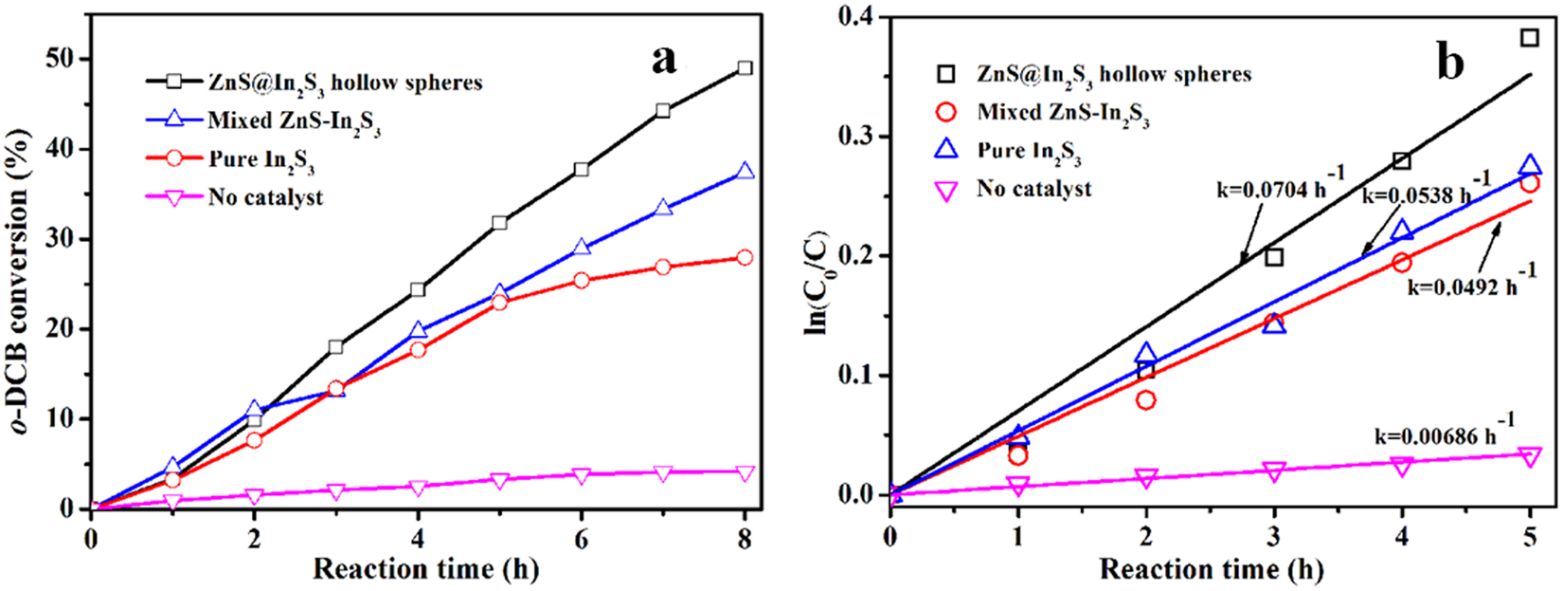
(**a**) Photocatalytic degradation and (**b**) kinetic curves for degradation of gaseous *o*-DCB over the obtained materials, respectively.

**Figure 8:**
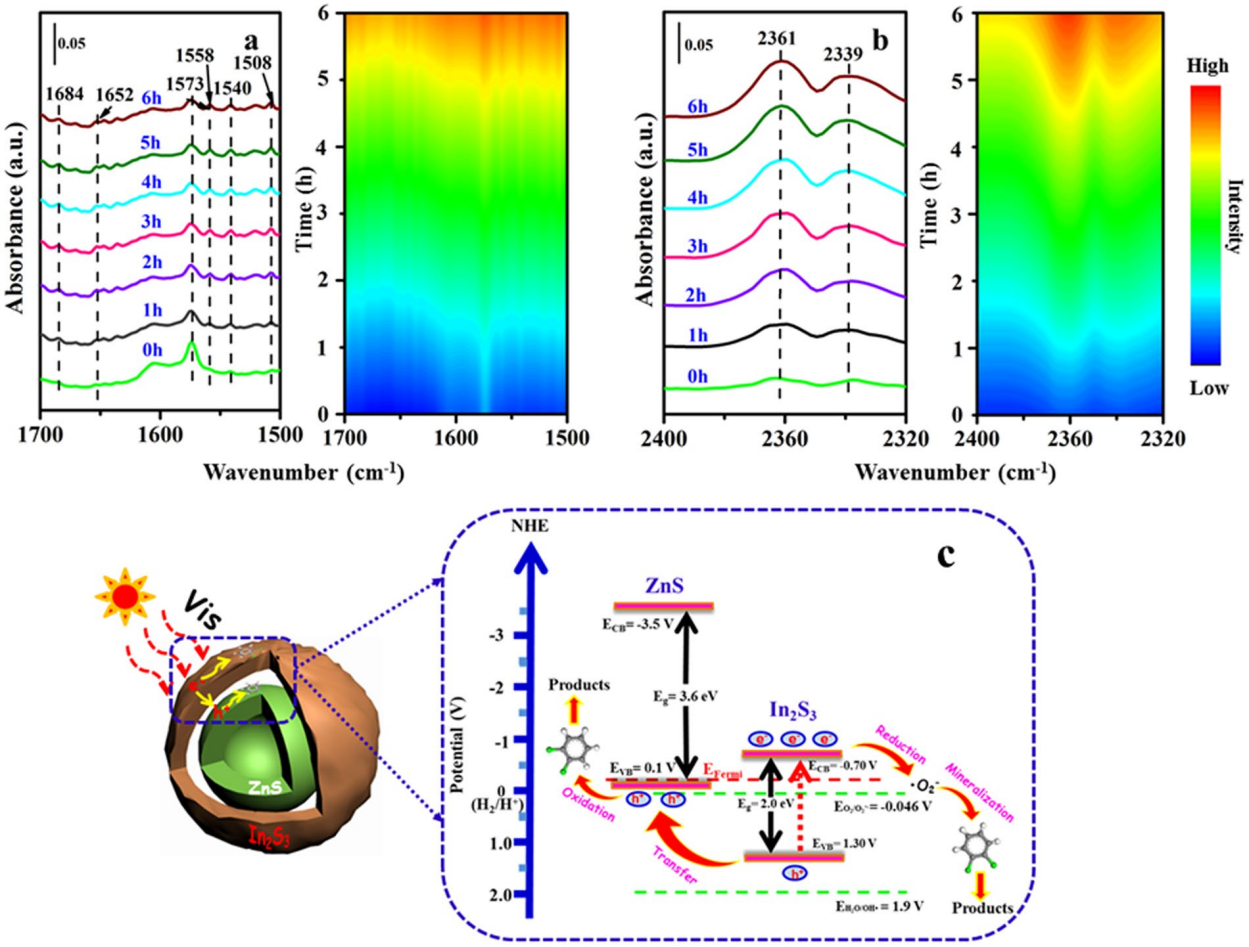
(**a**) *In situ* FTIR spectra in the different wavenumber regions, (**b**) their corresponding time-domain IR spectra, and (**c**) Schematic mechanism for gaseous *o*-DCB degradation over ZnS@In_2_S_3_ core-shell spheres.

Fig. 7a illustrates the photocatalytic performance for gaseous *o*-DCB degradation over all the samples obtained under visible light (λ > 400 nm). It was found that the substrate could hardly be decomposed as persistent organic pollutant without catalysts. When adding the visible-light responsive In_2_S_3_ photocatalyst, the degradation rate could reach 30% after 8 h reaction time for lower charge recombination rate and lower quantum efficiency. Compared with the simple mixed ZnS-In_2_S_3_, the ZnS@In_2_S_3_ core@shell hollow catalyst shows much higher degradation ratio (49%), suggesting the importance for having the hierarchical core-shell structure in photocatalytic reactions. Compared to other photocatalysts, the ZnS@In_2_S_3_ material shows the superiority for degradation of gaseous *o*-DCB under the identical and parallel conditions^17,23^ (Table S1, see Supporting Information). This is attributed to the following reasons: (1) The core@shell hollow structure possesses high specific surface area with the capability of modulating the index of refraction of light, which could provide more active sites and enhance the absorption ability; (2) The unique structure would increase the migration efficiency of photon-generated carriers, and then improve the quantum efficiency. In addition, the reaction kinetics over these catalysts were further investigated by Langmuir-Hinshelwood model^38^. As depicted in Figs 5h and 7b was selected as the targeted reaction time for some intermediates adsorbed on the surface of catalysts. It could be seen that all the degradation processes follow the pseudo-first-order fitting, and the kinetic constant over ZnS@In_2_S_3_ core@shell material (0.0704 h^−1^) is 1.3 and 1.4 times higher than that over the mixed ZnS-In_2_S_3_ (0.0538 h^−1^) and the pure In_2_S_3_ (0.0492 h^−1^), respectively. The results indicate that the degradation efficiency of gaseous *o*-DCB could be significantly enhanced with using the ZnS@In_2_S_3_ core@shell hollow structure. To explore the catalytic mechanism, the degradation experiments were conducted by *in situ* Fourier transform infrared (FTIR) spectroscopy and electron paramagnetic resonance (EPR) technique, which would be useful to investigate the reaction mechanism on the surface of catalysts^39^. As shown in Fig. 8a, some new peaks at 1684 and 1508 cm^−1^ were attributed to C=O stretching vibration of unsaturated aliphatic acid^40^, while those at 1558 and 1540 cm^−1^ were assigned to COO vibration of unsaturated aliphatic acid (formate and acetate)^41^. At last, the bands at 2361 and 2339 cm^−1^ (Fig. 8b) correspond to the C=O vibration of CO_2_, indicating that the gaseous *o*-DCB could be mineralized to CO_2_.

The possible degradation mechanism for gaseous *o*-DCB over ZnS@In_2_S_3_ core@shell catalyst was shown in Fig. 8c. When the composite material is irradiated by the visible light, the electrons of the outermost In_2_S_3_ shell would transfer from the VB to the CB for the narrow band-gap semiconductor, meanwhile, the In_2_S_3_ catalyst can create the same number of holes (h^+^) in the VB^42^. To these photo-induced electrons, the reduction reaction will occur with O_2_ adsorbed on the surface of In_2_S_3_, and then the important product, O_2_·- active species, is forming as more positive potential (E = −0.046 V) (see Supporting Information, Figure S2), which would further degrade the gaseous *o*-DCB to some products. On the other hand, the holes generated with strong oxidation ability will transfer to the VB of ZnS material with more positive potential (E = 1.30 V)^43^, and then react with the gaseous *o*-DCB to form other products^9^. In a certain extent, the characteristics of effective separation for photo-generated charges (e^−^ and h^+^) are beneficial to the enhancement of the photocatalytic performance of *o*-DCB degradation.

## Conclusions

In summary, the ZnS@In_2_S_3_ core@shell hollow composite was fabricated by the ion-exchange reaction between ZnS and In^3+^ ions in solution, and the formation mechanism is ascribed to the difference of solubility products (*K*_*sp*_) of ZnS and In_2_S_3_. The ZnS@In_2_S_3_ core@shell material exhibited the degradation rate of gaseous *o*-DCB of ~49% after 8 h reaction time under visible-light irradiation (λ > 400 nm), which is mainly attributed to the effective separation for photo-generated charges (e^−^ and h^+^). The ion-exchange method provides a new insight into the development of hollow photocatalysts with interior architecture.

## Methods and Materials

### Synthesis of Pure Zinc Sulfide (ZnS)

The synthesis process is similar to the previous reports^10^. In the typical synthesis, 0.734 g of Zn(AC)_2_ and 2 g of surfactant PVP were dissolved into 400 ml of ultrapure water and stirred for about 30 min to form a clear solution A. Meanwhile, 0.3 g of CH_3_CSNH_2_ (TAA) was also added into 200 ml of ultrapure water and stirred for about 15 min to form another clear solution B. The solution B was then slowly added into the solution A, and subsequently the mixture was continuously stirred in an oil bath at 100 °C for 2 h. At last, the products were collected by centrifugation and washed with absolute ethanol and ultrapure water for several times, and then dried overnight in a vacuum oven at 60 °C. The while powder was collected.

### Synthesis of Zinc Sulfide @ Indium Sulfide (ZnS@In_2_S_3_)

0.097 g of as-prepared ZnS sample was dissolved with ultrapure water to form a clear solution under ultra-sonication for about 5 min. Then, 0.3 g of In(NO_3_)_3_ was dissolved into the ZnS solution under continuously stirring for about 15 min, and later 0.075 g of TAA was added into the above solution for another 15 min stirring. Subsequently, the mixed solution was maintained at 80 °C for about 1 h under vigorously stirring and then naturally cooled down to room temperature. At last, the products were collected by centrifugation and washed with absolute ethanol and ultrapure water for several times, and then also dried overnight in a vacuum oven at 60 °C. The faint yellow powder was collected.

### Synthesis of Pure Indium Sulfide (In_2_S_3_) and Mixed ZnS-In_2_S_3_

The reaction process is the same as the synthesis of ZnS@In_2_S_3_ composite without adding ZnS solution. The mixed ZnS-In_2_S_3_ was fabricated by the manual milling method with the aforementioned ZnS and In_2_S_3_ particles.

### Materials Characterization

The X-ray diffraction (XRD) patterns were performed by an X-Ray diffractometer with Cu Kα radiation (Rigaku Corporation D/max-2400, Japan). The surface morphology of the samples was characterized by using a field emission scanning electron microscope (FESEM, Hitachi SU8010, Japan). The transmission electron microscopy (TEM) images were captured by using a FEI Tecnai G20 (USA). In addition, the chemical states and surface elements compositions were conducted by X-ray photoelectron spectroscopy (XPS, Thermo ESCALAB 250XI). The UV-Visible diffuse reflection spectra (DRS) were recorded by using an absorption spectrophotometer (JASCO, UV-550) from 200 to 800 nm. The specific surface area and pore distribution were investigated by nitrogen adsorption/desorption isotherms using an automated gas sorption analyzer (Autosorb-IQ, USA).

### Photoelectrochemical Measurements

The working electrode was prepared as follows: 0.01 g photocatalyst was mixed with ethanol under ultra-sonication for about 5 minutes, and the dissolved solution was then coated onto a piece of an indium-tin oxide glass (2 × 4 cm^2^) dropwise. The as-prepared electrode was dried and treated at 200 °C for about 1 hour in the N_2_ atmosphere. All electrodes had similar film thicknesses. Photocurrents and electrochemical impedance spectra (EIS) were measured by the electrochemical workstation (CHI760c) in a standard three electrode system using the prepared sample film as the working electrode, Ag/AgCl as the reference electrode, and the Pt flake as the counter electrode. Finally, a 500 W Xenon lamp with a UV-cut off filter (λ > 400 nm) was served as a visible-light source to irradiate the working electrodes, and 0.5 M Na_2_SO_4_ solution was used as the electrolyte.

### The Evaluation of Photocatalytic Degradation

To evaluate the photocatalytic performance of the as-prepared catalysts, the degradation efficiency of gaseous *o*-DCB was conducted in a home-built quartz reaction cell with the volume of about 130 mL^31^. First, the catalyst (0.02 g) pressed into the circular pieces was fixed to the holder and then the micro-reactor was sealed up immediately. Then, the gaseous pollutant was introduced into the reaction cell by injecting liquid *o*-DCB (5 µL) with a micro syringe. After an hour in the dark, the *o*-DCB was completely evaporated to gaseous pollution that could reach the adsorption equilibrium on the surface of catalysts. At that moment, the concentration of *o*-DCB was designated as the initial value, and the light (A 500 W Xenon lamp) equipped with a UV-cut off filter (λ > 400 nm) was turned on. Finally, *in situ* IR spectra were recorded by FTIR (Bruker VERTEX 70) in the region of 2400 and 1700 cm^−1^, and the concentrations of gaseous *o*-DCB were calculated in the reaction process according to the previous report^17^.

## Electronic supplementary material

Supporting Information
