# Supplementary material for: Preparation of ZnS@In2S3 Core@shell Composite for Enhanced Photocatalytic Degradation of Gaseous o-Dichlorobenzene under Visible Light
Source: Sci Rep. 2017 Nov 27;7:16396. doi: 10.1038/s41598-017-16732-4 (PMC5703877; doi:10.1038/s41598-017-16732-4)
Supplement: Supplementary file 1 — Supporting Information [file 41598_2017_16732_MOESM1_ESM.pdf]

Supporting Information

Preparation of $ZnS@In_2S_3$ Core@shell Composite for Enhanced Photocatalytic Degradation of Gaseous *o*-Dichlorobenzene under Visible Light

Baojun Liu[1,2], Xia Hu[1], Xinyong Li*[3], Ying Li[2], Chang Chen[2], Kwok-ho Lam*[2]

[1]*College of Resource and Environmental Engineering, Guizhou University, Guiyang 550025, China;*

[2]*Department of Electrical Engineering, The Hong Kong Polytechnic University, Hung Hom, Kowloon, Hong Kong*

[3]*State Key Laboratory of Fine Chemicals, Key Laboratory of Industrial Ecology and Environmental Engineering (MOE), School of Environmental Science and Technology, Dalian University of Technology, Dalian 116024, China*

*Corresponding authors: kokokh.lam@polyu.edu.hk; kokokhlam@gmail.com (K. H. Lam);
xyli@dlut.edu.cn (X. Y. Li).

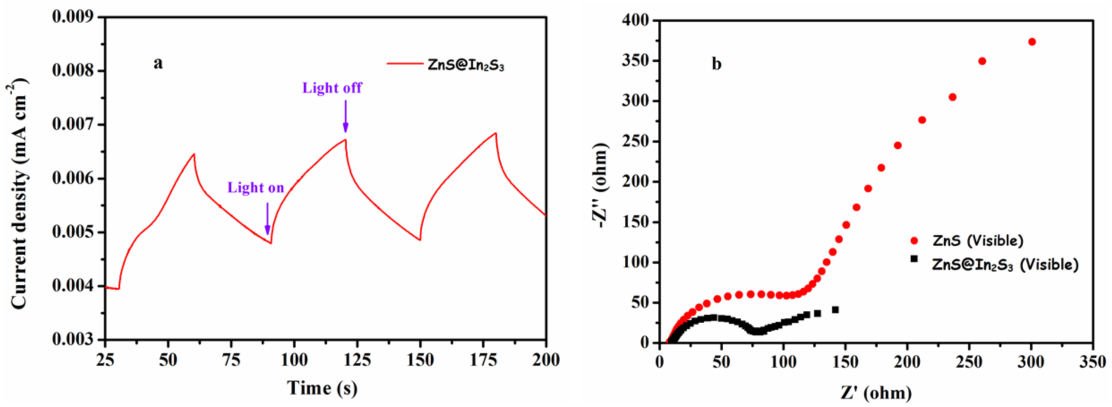

Figure S1: (a) Transient photocurrent response of $ZnS@In_2S_3$ in 0.5 M Na_2SO_4 mixed aqueous solution under visible-light irradiation. (b) Nyquist plots of ZnS and $ZnS@In_2S_3$ electrodes in 0.5 M Na_2SO_4 aqueous solution under visible-light irradiation.

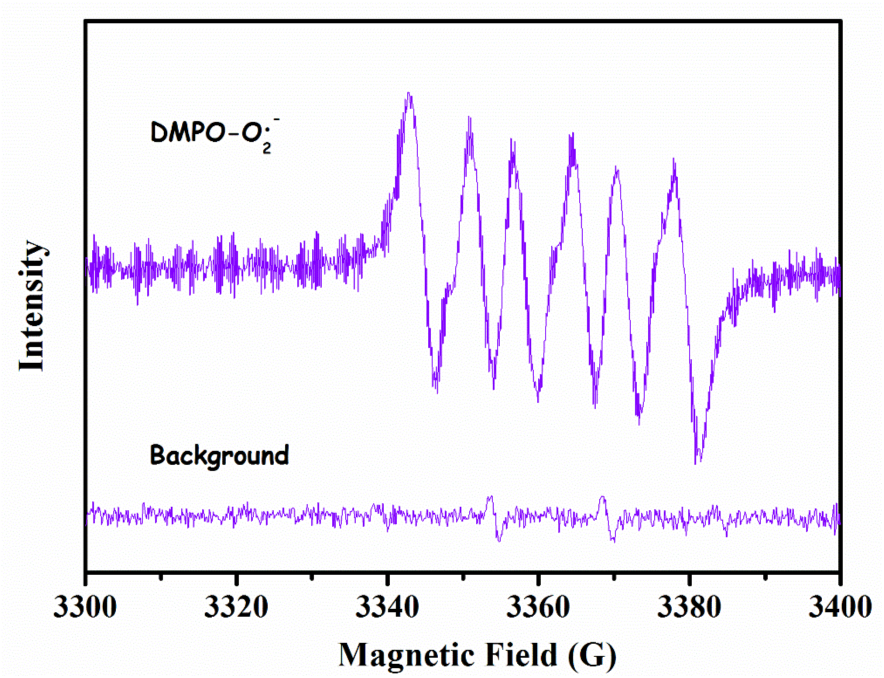

Figure S2: EPR spectra of the $ZnS@In_2S_3$ core@shell spheres methanol suspension solution after 40 s under visible light irradiation.

Table S1 Comparisons of rate constants and *o*-DCB conversions for using different catalytic materials under the identical and parallel conditions.

Materials	Rate Constants (h^{-1})	*o*-DCB Conversions (%)	Reference
**ZnS@In_2S_3**	0.0704	49.0	This work
**ZnS mixed with In_2S_3**	0.0492	37.4	This work
**Hollow V_2O_5**	0.101	45.2	1
**Commercial V_2O_5**	0.0517	18.4	1
**$AgInS_2/TiO_2$ (1: 1)**	0.0316	21.2	2
**$AgInS_2/TiO_2$ (1: 3)**	0.0416	26.6	2
**$AgInS_2/TiO_2$ (1: 5)**	0.134	50.4	2
**$AgInS_2/TiO_2$ (1: 10)**	0.0491	27.9	2

References

1 Liu, B. J. *et al*. Insight into the mechanism of photocatalytic degradation of gaseous *o*-dichlorobenzene over flower-type V_2O_5 hollow spheres. *J. Mater. Chem. A* **3**, 15163-15170 (2015).

2 Liu, B. J. *et al*. Preparation of $AgInS_2/TiO_2$ composites for enhanced photocatalytic degradation of gaseous *o*-dichlorobenzene under visible light. *Appl. Catal. B: Environ*. **185**, 1-10 (2016).
